# A comprehensive review of water quality indices for lotic and lentic ecosystems

**DOI:** 10.1007/s10661-023-11512-2

**Published:** 2023-07-08

**Authors:** Lazarus Katlego Mogane, Tracy Masebe, Titus A. M. Msagati, Esper Ncube

**Affiliations:** 1 College of Agriculture & Environmental Sciences, Department of Life and Consumer Sciences, University of South Africa, Roodepoort, Gauteng South Africa; 2 College of Science, Engineering & Technology, Institute for Nanotechnology & Water Sustainability, University of South Africa, Roodepoort, Gauteng South Africa; 3 School of Health Systems and Public Health, Faculty of Health Sciences, University of Pretoria, Tshwane, Gauteng South Africa

**Keywords:** Water quality index, Lotic, Lentic, Water quality parameters, Aquatic ecosystems

## Abstract

Freshwater resources play a pivotal role in sustaining life and meeting various domestic, agricultural, economic, and industrial demands. As such, there is a significant need to monitor the water quality of these resources. Water quality index (WQI) models have gradually gained popularity since their maiden introduction in the 1960s for evaluating and classifying the water quality of aquatic ecosystems. WQIs transform complex water quality data into a single dimensionless number to enable accessible communication of the water quality status of water resource ecosystems. To screen relevant articles, the Preferred Reporting Items for Systematic Reviews and Meta-Analyses (PRISMA) method was employed to include or exclude articles. A total of 17 peer-reviewed articles were used in the final paper synthesis. Among the reviewed WQIs, only the Canadian Council for Ministers of the Environment (CCME) index, Irish water quality index (IEWQI) and Hahn index were used to assess both lotic and lentic ecosystems. Furthermore, the CCME index is the only exception from rigidity because it does not specify parameters to select. Except for the West-Java WQI and the IEWQI, none of the reviewed WQI performed sensitivity and uncertainty analysis to improve the acceptability and reliability of the WQI. It has been proven that all stages of WQI development have a level of uncertainty which can be determined using statistical and machine learning tools. Extreme gradient boosting (XGB) has been reported as an effective machine learning tool to deal with uncertainties during parameter selection, the establishment of parameter weights, and determining accurate classification schemes. Considering the IEWQI model architecture and its effectiveness in coastal and transitional waters, this review recommends that future research in lotic or lentic ecosystems focus on addressing the underlying uncertainty issues associated with the WQI model in addition to the use of machine learning techniques to improve the predictive accuracy and robustness and increase the domain of application.

## Introduction

Freshwater resources play a pivotal role in sustaining life and meeting various domestic, agricultural, economic, and industrial demands. However, there are increasing concerns about water security and quality, especially in arid and semi-arid regions of the world (Aragaw & Gnanachandrasamy, 2021; Busico et al., 2020). The concerns emanate from the continuous pollution of water resources from anthropogenic, industrial, and agricultural sources which have become a serious environmental issue, requiring serious strategies for constant monitoring and enforcement of regulatory policies to sustain such ecosystems (Gupta et al., 2009; Mahlathi et al., 2016; El-Batrawy et al., 2018; Nagy-Kovacs et al., 2019; Sandhu et al., 2019). It is also important to realise that the degrading water quality decreases the portion of available safe and clean water, while the demand and dependence on water by humans and animals remain the same (Young & Beck, 1974; Peters & Meybeck, 2000; Kanakoudis & Tsitsifli, 2020). However, in the case of humans, they have adapted survival mechanisms for dealing with polluted water resources through treatment processes for various uses, while animals do not have such options (Rangeti et al., 2015). As it stands the current situation has become such that rivers, streams, and dams are not only reliable sources of freshwater supply but also the disposal points of either untreated or partially treated wastewater effluents (Bartram & Balance, 1996; Das & Acharya, 2003; Tukura et al., 2009; Edokpayi et al., 2017). This has led to the mass contamination of water resources and has affected the normal functioning of aquatic ecosystems in many places (Kumarasamy & Macholo, 2018). Therefore, monitoring water quality to ensure the safety of consumers and the ecosystem has been an issue of paramount importance. To ensure that the monitoring and safeguarding of water resources are effective, it is imperative to put strategies in place to understand, improve, and mitigate such effects. As such, various water quality indices (WQIs) have been devised.

A water quality index (WQI) model is a tool that converts large water quality data into a single value called the index score. The WQI model is comprised of five stages of development which involve parameter selection, generation of sub-index functions, the establishment of parameter weights, aggregation of sub-index values and determination of classification schemes. The importance of WQIs for the evaluation of water quality is highlighted by the number of studies that seek to put to light the limitations (Abbasi & Abbasi, 2012; Sutadian et al., 2016; Uddin et al., 2021; Gupta & Gupta, 2021), and possible solutions (Malek et al., 2022; Uddin et al., 2022a, b, c, 2023a, b) in order to improve the accuracy, robustness, reliability and wide acceptability. The earlier models of WQIs involved several subjective methods such as the Delphi technique and expert opinions in the development stages, especially parameter selection. This has been reported by most studies as a source of uncertainty and contributed to low model acceptability.

The attractive aspect of using water quality indices in water resources management is that they present a qualitative method of aggregating or summarising water quality datasets from different parameters in a simpler, easier and more understandable way (Couillard & Lefebvre, 1985; Cude, 2001; Tanner et al., 2011; Hoseinzadeh et al., 2015; Barakat et al., 2018). Various water quality indices have been developed since 1965, with each customised based on the relevant water resource in a specific region (Horton, 1965). Most of these indices tend to differ based on the water quality parameters used to develop them, the calculation algorithm and the scale used to rate the water quality (Tyagi et al., 2013; Feng et al., 2016, Malek et al., 2022, Uddin et al., 2023c). These indices simplify complex water quality data for political decision-makers, water resource managers who are not technically inclined and the public (Mladenovic-Ranisavljevic & Žerajic, 2017). Beyond simplifying complex water quality data, WQIs have been used as vital tools to gain knowledge about pollutants and their transport processes and predict the quality of water resources (Kumarasamy & Macholo, 2018).

Although there is not any globally standardised or harmonised methodology for developing a WQI (Sutadian et al., 2016), water quality index usage in the assessment of surface water has been well documented (Banerjee & Srivastava, 2009; Alobaidy et al., 2010; Massoud, 2012; Sutadian et al., 2016; Sener et al., 2017). However, a limited number of systematic reviews on the development and use of WQIs have so far been published and are available in the open literature, and they include those that deal with exploring the different types of WQIs (Gupta & Gupta, 2021; Uddin et al., 2021), assessing steps in their development, advantages (Sutadian et al., 2016) and disadvantages (Uddin et al., 2021; Chidiac et al., 2023). For example, Lumb et al. (2011) reviewed other WQI models developed between 1960 and 2010, where they demonstrated the importance of the steps used in the formulation of the WQI. In addition, Sutadian et al. (2016) reviewed 30 models of WQIs, the country where they were developed and applied, and more recently, a review of the accuracy of commonly used WQIs by Uddin et al. (2021). The available WQIs used to evaluate surface water quality are based on physical and chemical parameters and very few microbial pathogens such as *Escherichia coli*, faecal coliforms and total coliforms.

Notwithstanding, the introduction and wide application of machine learning tools have significantly evolved the architecture of the modern WQI models (Gazzaz et al., 2012; Najafsadeh et al., 2021; Malek et al., 2022; Uddin et al., 2022b). Many WQI models are designed for a specific domain of application such as the Oregon index (Dunnette, 1979), National Sanitation Foundation (NSF) index, Malaysian index (DoEM, 2002), West Java index (Sutadian et al., 2018), etc., and precisely to address a specific regional water quality problem such as the Irish Water Quality Index (IEWQI) (Uddin et al., 2023a). Although selected WQI models have been customised for application in other regions such as the NSF and the Scottish Research Development Department (SRDD), the domain of application has always been the same. However, the success of the IEWQI especially with reducing uncertainty while being applied in multiple domains should serve as a useful benchmark for future WQI model developers. The present study seeks to investigate the possibility of applying one WQI model to assess both lotic and lentic systems. In addition, the authors would like to present workable solutions to allow the seamless application of WQI models in both lotic and lentic systems with significant efficacy.

## Method and approach of review

The current study followed the Preferred Reporting Items for Systematic Reviews and Meta-Analyses (PRISMA) guidelines (Moher et al., 2009). PRISMA is an evidence-based system composed of a set of items for reporting systematic reviews and meta-analysis. By primary design, PRISMA is used to report review studies that evaluate the effects of interventions such as aetiology, prevalence, diagnosis or prognosis. However, the methodology can also be employed in review studies that have an objective other than that of evaluating interventions. The method is described in full by Page et al. (2021).

## Review question, inclusion and exclusion criteria for articles, and criteria for articles and models

As discussed before, WQI models are primarily developed for specific regions and to solve regional water challenges. It is only after it has demonstrated reliable performance and less uncertainty that other regions will attempt to customise and use that model for their own water quality challenges. However, if a model has been optimised to only evaluate the water quality of a river, lake or marine system, the pertinent question to is, “can that WQI model be used to assess the quality of both lotic and lentic ecosystems with equal efficacy?”

As part of the article filtering process, the present study devised the inclusion or exclusion criteria for articles found in all accessed databases. The inclusion criteria for relevant articles included articles where WQIs were developed through four stages or steps: parameter selection, the transformation of parameters to a standard scale, the weighting of parameters and aggregation and used for the general assessment of water quality. The exclusion criteria involved articles that applied an existing WQI and used a WQI model with a specific water assessment use. Furthermore, only peer-reviewed original articles were included in the final review synthesis.

## Articles search strategy

**Fig. 1:**
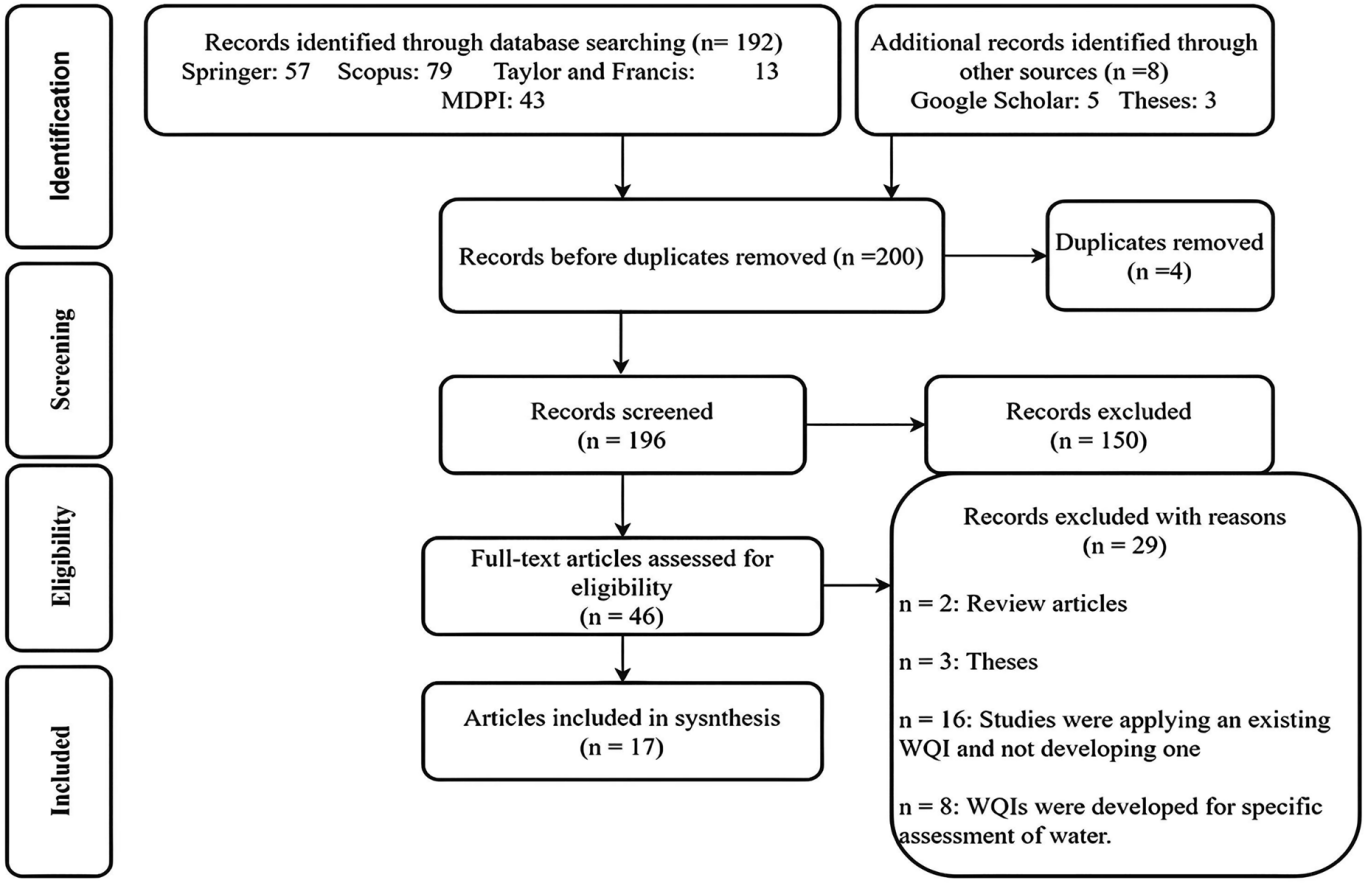
PRISMA flow diagram of searching, screening and article selection

The article search parameters were defined to address the objectives of this study. The key phrases for the searches included the following: “water quality index for lentic systems”, “water quality index for lotic systems” and “water quality index development”. The “AND” Boolean operator was applied to all the search phrases to narrow the search results. Five article databases ((Springer: https://link.springer.com/); (MDPI: https://www.mdpi.com/); (Scopus: https://www.scopus.com/); (Taylor and Francis Online: https://www.tandfonline.com/); and (Google Scholar: https://scholar.google.com/)) were used for article search. The following Fig. 1 is a representation of the article filtering process that ends with 17 articles used for the final synthesis. These 17 articles are also presented in detail in Table 1.

**Table 1:** Overview of included studies, appraisal of the stages of development, system of application and where and when studies were first published

Name of WQI	Selected parameters and method of selection	Transformation	Weighting	Aggregation method	System of application	Country of first application
Liou’s index (Liou et al., 2004)	Literature and principal component analysis (PCA)	Parameters are taken directly as sub-indices using permissible limits	PCA	Geometric mean	Lotic systems	Taiwan
	9 parameters: DO, BOD_5_, ammonia nitrogen, SS, turbidity, FC, temperature, toxicity and pH					
Pesce index (Pesce & Wunderlin, 2000)	GEMS/Water UNEP Program recommendations	Ranking using permissible limits	Researchers’ experience	Arithmetic mean	Lotic systems	Argentina
	20 parameters: ammonia, BOD_5_, calcium, chloride, COD, DO, hardness, magnesium, nitrates, nitrites, oil and grease, pH, orthophosphate, solids: dissolved, solids: total, sulphates, surfactants, temperature, total coliforms and turbidity.					
Hanhindex (Hanh et al., 2011)	PCA	Piecewise linear membership rating functions	No weighting	Linear product power and linear sum power	Lotic and lentic systems	Vietnam
	8 parameters: DO, turbidity, SS, COD, BOD_5_, ammonium nitrogen, orthophosphate and total coliforms					
Indian pollution index (Sargaonkar & Deshpande, 2003)	Indian water resources quality objectives	Rating curves with permissible limits	No weighting	Arithmetic mean	Lotic systems	India
	13 parameters: turbidity, pH, colour, DO, BOD_5_, TDS, hardness, chloride, sulphate, nitrate, total coliforms, arsenic and fluoride					
Prati index (Prati et al., 1971)	Through literature	Linear and parabolic functions	No weighting	Arithmetic mean	Lotic systems	Italy
	13 parameters: pH, DO, BOD, COD, SS, ammonia, nitrates, chlorine, iron, Mn, alkyl benzene sulphonates and carbon chloroform extract.					
NationalSanitation Foundation (NSF) (Brown et al., 1970)	Delphi technique	Delphi technique	Delphi technique	Originally arithmetic but later changed to multiplicative (Brown et al., 1973)	Lotic systems	USA
	11 parameters: DO, pH, BOD_5_, temperature, total phosphorus, nitrates, turbidity, total solids, pesticides, toxicity and faecal coliforms					
Canadian Council of Ministers of the Environment (CCME) index (CCME, 2001)	Not set, can be customised by the user	Standard guidelines or water resource quality objectives	No weighting	Sum root of squares	Lotic and lentic systems	Canada
The Scottish Research Development Department (SRDD) index (SRDD, 1976)	Delphi technique	Delphi technique	Delphi technique	Arithmetic mean	Lotic systems	Scotland
	10 parameters: DO, BOD_5_, pH, phosphate, SS, temperature, conductivity, total oxidised, free, and saline ammonia and *E. coli*					
Malaysian index (DoEM, 2002)	Expert panel opinion	Parameters are taken directly as sub-indices	Unequal weighting through expert opinions	Arithmetic mean	Lentic systems	Malaysia
	6 parameters: pH, DO, COD, ammoniacal nitrogen, SS and BOD					
Hallockindex (Hallock, 2002)	Delphi technique	Rating curves	Delphi technique	Arithmetic mean	Lotic systems	Washington State
	8 parameters: DO, pH, total nitrogen, total phosphorus, total suspended solids, turbidity and faecal coliforms.					
Said’s index (Said et al., 2004)	Environmental importance	Parameters are taken directly as sub-indices	Not required	Mathematical function	Lotic systems	Florida State
	5 parameters: DO, turbidity, total phosphorus, specific conductivity and faecal coliforms					
Universal Water Quality index (UWQI) (Banda & Kumarasamy, 2020)	Expert opinion	Parameter relative environmental importance	Target water quality ranges (TWQR)	Weighted arithmetic	Lotic systems	South Africa
	13 parameters: ammonia, calcium, chloride, chlorophyll a, conductivity, fluoride, hardness, magnesium, manganese, nitrate, pondus hydrogenium, sulphate and turbidity					
West -Java WQI (Sutadian et al., 2018)	Availability of monitoring data against standards; statistical assessment.	Linear scaling for temperature and mathematical function for other parameters	Evaluation of expert opinion with analytical hierarchy process (AHP)	Multiplicative	Lotic systems	Indonesia
	13 parameters: temperature, SS, COD, DO, nitrite, total phosphate, detergents, phenols, chloride, zinc, lead, mercury and faecal coliforms.					
Dinius index (Dinius, 1987)	Delphi technique	Rating functions	Expert opinion	Multiplicative	Lotic systems	Alabama State
	12 parameters: DO, BOD_5_, total coliforms, *E. coli*, alkalinity, hardness, chloride, specific conductance, pH, nitrate, temperature and colour					
Houseindex (House, 1989)	Interviews with water stakeholders	Rating curves	Questionnaire survey	Arithmetic mean	Lotic systems	UK
	9 parameters: DO, ammoniacal nitrogen, SS, pH, temperature, BOD_5_, nitrates, chlorides and total coliforms					
River Ganga Index of Ved Prakash et al. (Abassi & Abassi, 2012)	Delphi technique	Rating curves from expert opinion data	Relative weight assignment	Weighted arithmetic mean	Lotic systems	India
	4 parameters:					
	DO, BOD, pH and faecal coliforms					
IrishWater Quality Index (IEWQI) (Uddin et al., 2023a)	Random forest machine learning algorithm	Interpolation rescaling functions	Random forest machine learning and rank sum mathematical function	Quadratic mean	Lotic and lentic systems	Ireland
	9 parameters: salinity, DO, BOD_5_, pH, water temperature, transparency, total oxidised nitrogen, dissolved inorganic nitrogen, molybdate reactive phosphorus.					

## The typical steps for developing a WQI

The development of a WQI involves five steps which include:

1. Parameter selection is the process where water quality parameters or variables are selected for inclusion based on their importance in a specific region. This step involves the use of the Delphi technique (Mladenović-Ranisavljević & Žerajić, 2017), a panel of experts to give professional judgement (House, 1980), statistical methods (Sutadian et al., 2018; Guo et al., 2019; Parween et al., 2022) and machine learning techniques (Uddin et al., 2023a).
2. Transformation to a standard scale includes converting the various parameter units to unitless sub-indices. Various methods have been applied among existing WQI models which involve the use of rating functions (Fathi et al., 2022), interpolated functions (Parween et al., 2022; Uddin et al., 2022a, 2023a), expert opinions (Dinius, 1987) and taking parameter concentrations directly as sub-index values (Liou et al., 2004; Said et al., 2004).
3. Establishing parameter weights involves assigning weightage to the parameters based on their overall relative importance to the assessment (Uddin et al., 2023a). Various methods exist in literature including subjective techniques such as Delphi technique (Dadolahi-Sohrab et al., 2012), expert opinions (Sutadian et al., 2018), mathematical functions such as the rank sum technique (Uddin et al., 2023a) and statistical methods such as AHP (Sutadian et al., 2018).
4. Aggregating the transformed parameters to produce the final index value includes computing a single comprehensive index value by combining the sub-index and assigned weightings. Different aggregation methods exist in literature and include three main categories: additive or arithmetic (Horton, 1965; Prati et al., 1971; House, 1989), multiplicative (Asadollah et al., 2021; Parween et al., 2022) and logical or a combination of arithmetic and multiplicative (Almeida et al., 2012; Dadolahi et al., 2012).
5. Water quality index score and classification schemes. These schemes consist of five classification categories (Horton, 1965; Brown et al., 1970; CCME, 2001; Sutadian et al., 2018) and sometimes less (DoEM, 2002; Uddin et al., 2022a) or more categories (SRDD, 1976).

**Fig. 2:**
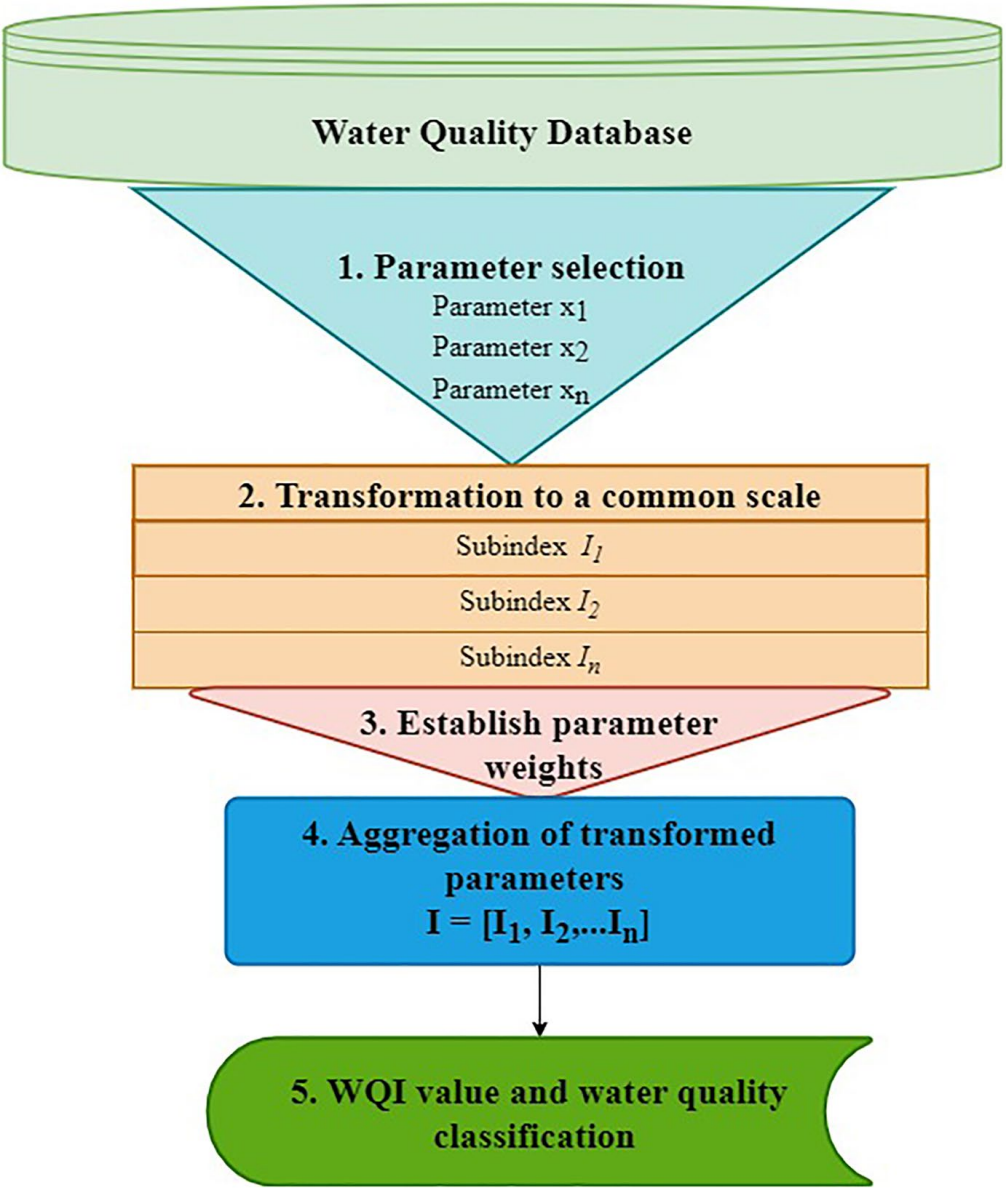
The general WQI development steps

These steps are represented in Fig. 2 (Abbasi & Abbasi, 2012; Sutadian et al., 2018; Uddin et al., 2021, 2023a).

The development steps are further discussed in sequence. This discussion is followed by an in-depth look at some of the most used WQIs.

### Parameter selection

The consensus is that it is impossible to continuously monitor all water quality parameters because of financial implications and time. As such, the most critical parameters are considered instead. This stage is the most challenging because omitting parameters may lead to a loss of information and misinformation about water quality (Rangeti et al., 2015). This stage is also prone to subjectivity and uncertainty (Sutadian et al., 2016; Uddin et al., 2021, 2023a). It is therefore recommended that the opinions of local water quality stakeholders, experts and government officials be considered. The original WQI by Horton (1965) used a committee of experts to deliberate whether a particular variable could be added. This was later criticised by Joung et al. (1979) because experts disagreed on the relevance and relative significance of parameters. These disagreements opened loopholes for criticism by another committee of experts. The Delphi technique is another method that considers the expert contributions, only there is no debate, but a series of questionnaires sent to the participants without them converging in one area (Delbecq et al., 1975). As Lohani and Todino (1984) argued, these approaches of parameter selection are highly subjective because their professional backgrounds prejudiced the judgment of these experts. This led to the suggestion of complex statistical methods such as factor analysis (FA) and PCA, which are more robust and eliminate the compelling issue of biases (Lohani & Todino, 1984; Jolliffe, 2005; Rangeti et al., 2015; Sutadian et al., 2018). Despite the accuracy of these statistical methods (Kumar et al., 2019; Ma et al., 2020; Chakravarty & Gupta, 2021; Parween et al., 2022), their adoption for use has been less because of the complex statistics they come with. Many studies (Medeiros et al., 2017; Sutadian et al., 2018) have continued to use expert judgment for parameter selection.

FA and PCA have been recommended for use in modelling studies (May et al., 2011), especially with intelligent learning systems such as artificial neural networks (ANNs) (Singh et al., 2009; Rangeti et al., 2015). However, recent research has reported that these existing methods contribute significantly to the unreliability of a model and the inappropriateness of the selected parameters (Uddin et al., 2021, 2023b). In a recent study, the authors compared different methods for optimising parameter selection which included filter, wrapper, and embedded methods. The authors reported that embedded-based methods such as random forest and extra tree and filter-based mutual information methods outperform the commonly used filter-based methods such as PCA and Pearson correlation. In addition, the authors reported that the usage of these methods helped to improve model performance by reducing model uncertainty due to less robust parameter selection methods (Uddin et al., 2023b).

**Fig. 3:**
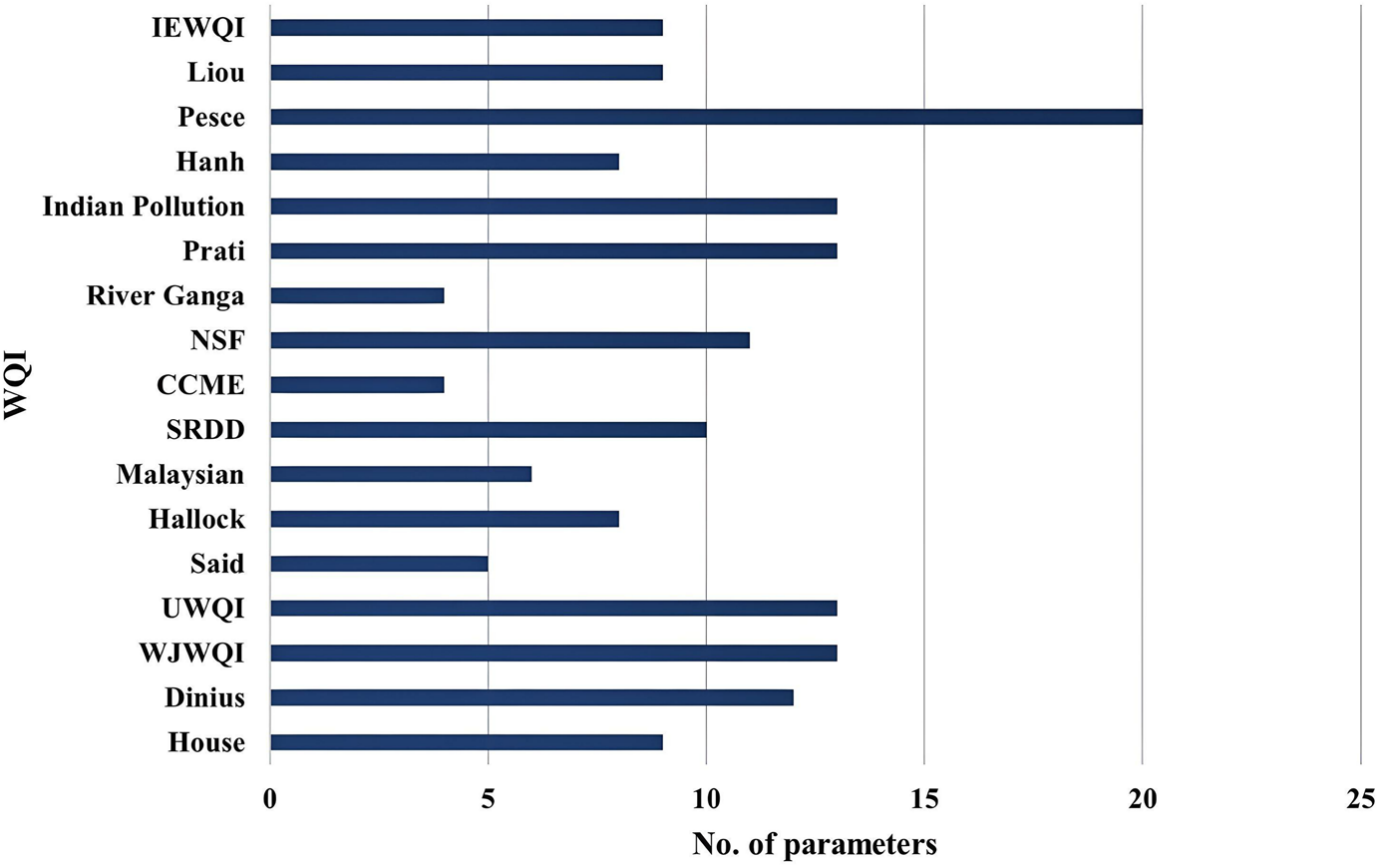
Summary of the number of parameters used per WQI included in the study

**Fig. 4:**
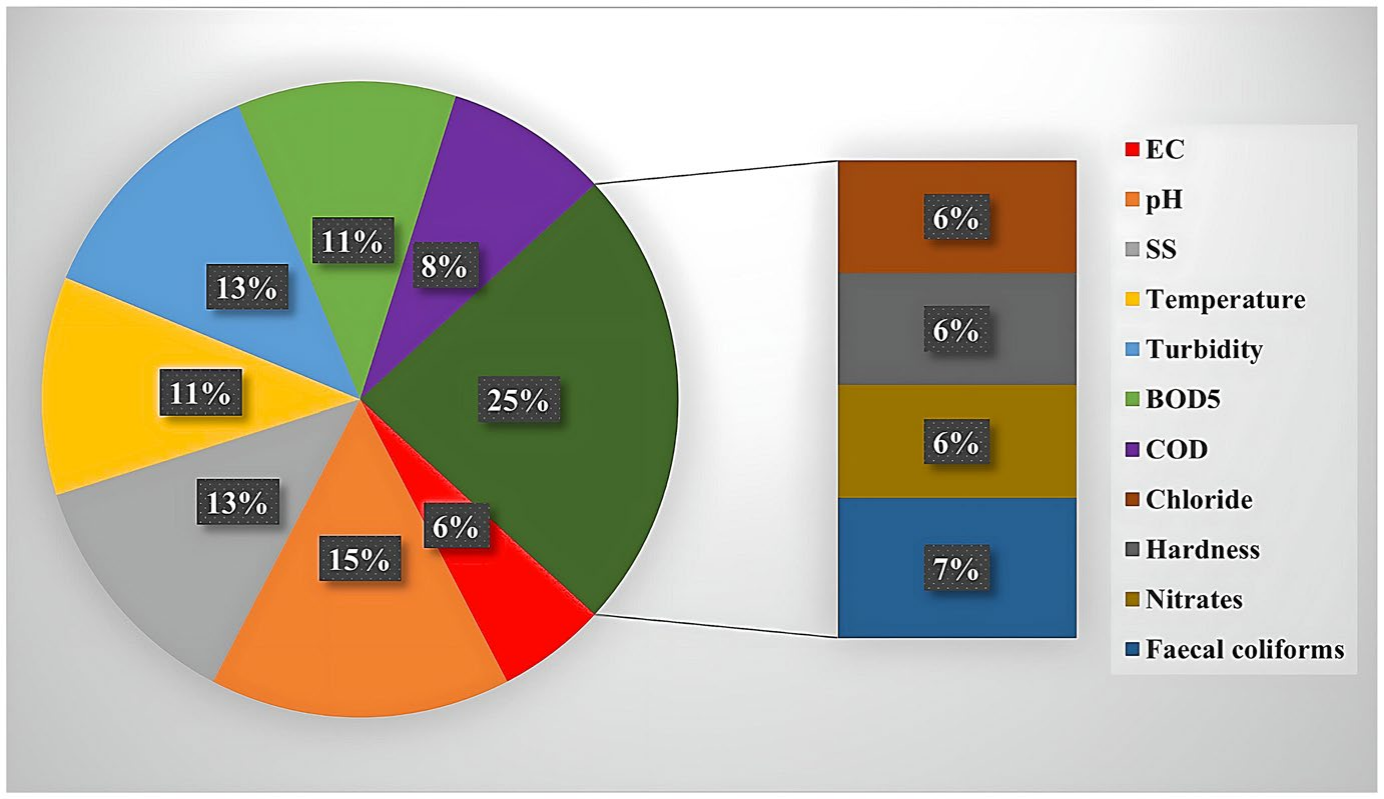
Percentage of the frequency of use for individual water quality parameters in all selected WQIs

Among the 17 WQIs assessed, the number of selected parameters was different. The Pesce index had the highest number of parameters (20), while the River Ganga index of Ved Prakash et al. had only four parameters (Fig. 3) The CCME requires a minimum of four parameters, but the number is user dependent. Figure 3 presents a summary of the number of parameters used for each analysed WQI model, and Fig. 4 illustrates the frequency of use for each parameter among the 17 studied WQI models.

Water quality is a collective term for water’s physical, chemical and biological quality. As such, any assessment of water quality must include all three categories of water quality. Among the analysed WQIs, physical parameters were mainly used (56%) as compared to chemical parameters (37%) and biological parameters (7%) (Fig. 4). This denotes that some WQIs do not include all categories of water quality.

### Transformation to a standard scale

The part of parameter transformation aims to convert the different selected parameters to a single dimensionless scale. This is important because their different scales will only be possible to aggregate the parameters together. For example, turbidity is presented in nephelometric turbidity units (NTUs), while manganese (Mn) and iron (Fe) in milligrams per litre (mg/L) (Rangeti et al., 2015; Sutadian et al., 2016). Moreover, different parameters have different ranges of acceptable fit-for-use standards. For instance, dissolved oxygen (DO) seldom gets readings beyond 0–12 mg/L, whereas sodium can be between 0 and 1000 mg/L (Abbasi & Abbasi, 2012). In essence, different parameters have different impacts concerning concentration. Parameter transformation eliminates the units of the various parameters and produces a new scale that is without dimension but two end-points (Ott, 1978; Dunnette, 1979), one with the lowest endpoint representing unacceptable values and the other with the highest end and representing acceptable quality (Richardson, 1997). While this step is crucial for aggregation, a few WQI models, such as the CCME, do not consider transforming parameters but use multivariate statistical procedures to aggregate the actual values of the parameters. Said et al. (2004) also developed a new WQI and proposed a mathematical equation for directly standardising the index without generating sub-indices. To create sub-index values, rating functions are developed. These curves are mathematical relationships between a parameter’s concentration value and the water quality. Common ways to determine sub-index functions include using water quality standards, expert judgment and statistical methods (Harkins, 1974; Lohani & Todino, 1984; Sutadian et al., 2016; Uddin et al., 2021). Despite the wide use of mathematical functions, many researchers have reported that sub-index functions are a source of model uncertainty (Sutadian et al., 2016; Gupta & Gupta, 2021; Uddin et al., 2021, 2022b, 2023a). As such, a recent study developed a hybrid method that involved the use of linear interpolation rescaling functions with threshold water quality guidelines to ameliorate model uncertainty (Uddin et al., 2022a).

**Fig. 5:**
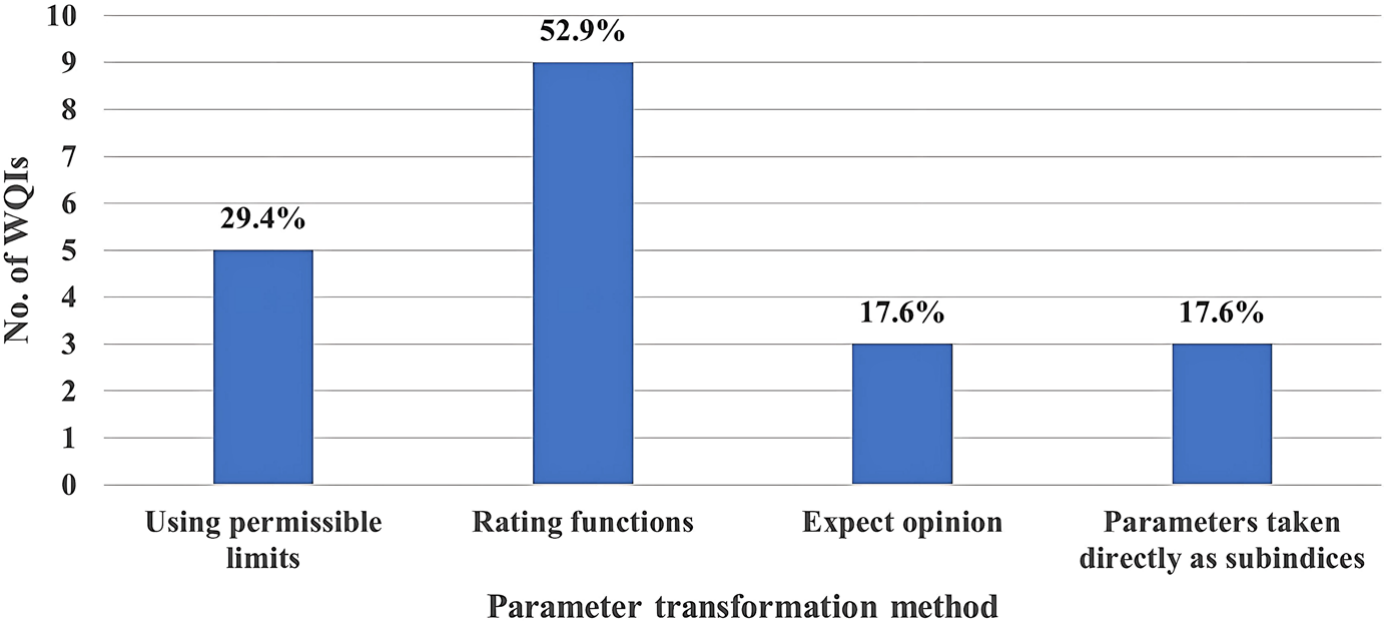
Usage of parameter transformation method among the analysed WQIs

Among the analysed WQIs, rating functions were used (52.9%) for transformation. The Liou, Indian pollution, and River Ganga indices used various methods. Of the analysed WQIs, 29.4% used standard permissible limits, while very few indices (17.6%) used expert opinions (Fig. 5). This is presented in Fig. 5 in sequence which illustrates the frequency of use of transformation methods among the analysed WQIs.

### Establishing parameter weights

The weight of a parameter is assigned based on its relative importance and influence on the overall value of the water quality index (Dzwairo et al., 2012). For purposes of credibility, well-formulated techniques are used. To correctly assign a weight, one needs to have knowledge of the parameter, its threshold standard limits, and its normal concentration range in a particular water resource (Kumar & Dua, 2009). If the parameters are equally important, equal weights are assigned, and unequal weights are allocated if the parameters have lesser or greater importance. Sapkal and Valunjkar (2013) considered, along with the latter, treatment methods of parameters. For example, if a parameter requires advanced treatment methods for removal, a lower weighting is assigned, and a higher weighting is allocated if the parameter requires conventional treatment. Other methods to assign parameter weights include participatory-based approaches, which may involve water quality experts or managers, policymakers, environmental protection agencies and other key stakeholders. However, the Delphi method and analytical hierarchy process (AHP) have been widely used (Kodikara et al., 2010, Sutadian et al., 2018). The AHP uses pairwise comparison matrices where the respondents specify their preferences based on other choices. In recent years, studies have reported these methods to be significant sources of uncertainty due to inappropriate weight estimation (Uddin et al., 2021, 2022a). Uddin et al. (2021) went further to document the sources of eclipsing and uncertainty for different WQI models. As such, different robust techniques such as the rank sum method (Uddin et al., 2022c), random forest machine learning (Uddin et al., 2023a) and extreme gradient boosting (XGB) machine learning (Uddin et al., 2022a) have been developed to ameliorate uncertainty in WQI models. In addition, recent studies have used machine learning approaches such as XGB, SVM, LSBoost and DNN to estimate model performance with regards to uncertainty (Uddin et al., 2023b).

### Aggregation of transformed parameters to produce the final index

Most aggregation methods are possible and have been applied to obtain a single value representative of the overall quality of water (Abassi & Abassi, 2012; Fu & Wang, 2012). This is the most crucial step in the whole procedure because of the potential loss of information and data distortions such as ambiguity, eclipsing and rigidity (Ball & Church, 1980; Couillard & Lefebvre, 1985; Abassi & Abassi, 2012). Ambiguity occurs when the overall index value is above the limit value when none of the considered individual parameter scores does not exceed the limit. At the same time, eclipsing occurs when the overall index does not exceed standard limits, but one or more of the considered individual parameters exceed the set limits. Rigidity, however, occurs when a need arises to add more parameters to an existing index to address new water quality concerns and the model does not allow such addition. There are three most common categories of aggregation methods: (i) additive or arithmetic, (ii) multiplicative or geometric, and (iii) logical. These are documented by Abassi and Abassi (2012) and Uddin et al. (2021). Additive or arithmetic aggregation methods involve combining the transformed parameters through summation. This method characterised the early days of WQI models (Horton, 1965; Brown et al., 1970; Prati et al., 1971; Ott, 1978). The weighted arithmetic mean is the most used additive aggregative method. Multiplicative or geometric aggregation methods, such as the indices of Walski and Parker (1974) and Dinius (1987), combine the transformed parameters through product operation, while logical techniques, such as the Smith index, combine the transformed parameters through a logical operation such as minimum and maximum (Smith, 1990). The additive and multiplicative aggregation methods have been identified as sources of eclipsing (Uddin et al., 2021). However, the logical aggregation approach was developed specifically to solve the eclipsing problem (Smith, 1990). The eclipsing data distortion during aggregation results in the overall index value overestimation or underestimation as observed by Uddin et al. (2022a, 2023a). Moreover, studies have achieved success using machine learning approaches such as ANN (Gazazz et al., 2012; Uddin et al., 2022b).

**Fig. 6:**
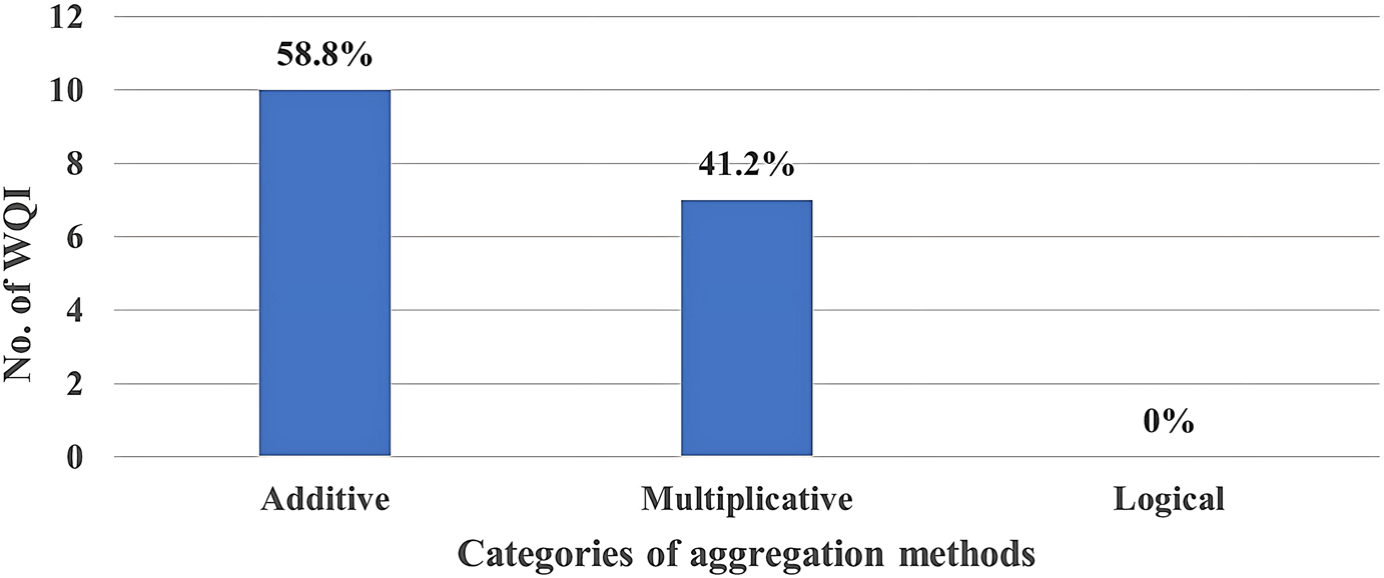
Aggregation category usage among the analysed WQIs

Most (58.8%) of the analysed WQIs used additive methods, while 41.2% used multiplicative methods (Fig. 6) to resolve the final index.

#### Categories of aggregation methods

(i) Additive
  The additive method is the most common one recorded in the available literature. The method involves using summation to combine the transformed values of the parameters to produce the index value (Abbasi & Abbasi, 2012). Indices of general water quality assessment using the additive method include Prati index (Prati et al., 1971) and Pesce index (Pesce &Wunderlin, 2000). One of the most used additive methods is the weighted arithmetic mean. The weighted arithmetic mean measures the central tendency of a set of observational data when not all observations have the same importance. This method has been used to aggregate transformed parameters of existing water quality indices, such as that of Brown et al. (1970), Dunnette (1979) and Sargaokar and Deshpande (2003). It has been recognized for offering simplicity, where the final value of the index is calculated by summating the weighted transformed parameters. Although this method deals away with eclipsing (Rangeti et al., 2015), it has been criticised for lacking sensitivity (Liou et al., 2004; Juwana et al., 2012; Sutadian et al., 2016). An example of this aggregation method is shown in Eq. 1:
  1 $$\mathrm{WQI}= \textstyle\sum^{\mathrm{n}}_{i\,=\,1}\mathrm{Q}i.\mathrm{W}i$$
  where *Q*_*i*_ = sub-index/transformed parameter *i*, *n* = number of transformed parameters and *W*_*i*_ = weight of the transformed parameter.
(ii) Multiplicative
  The multiplicative aggregation method involves combining the sub-index values through a product operation. In this category, the weighted geometric mean is the most used method. Indices of Dinius (1987), Walski and Parker (1974), Liou et al. (2004) and the SRDD (1976) which are for the general assessment of water quality have employed this model. The weighted geometric mean, in comparison to the weighted arithmetic mean, is more viable and unbiased (Landwehr & Deininger, 1974; Joung et al., 1979) and has been used as an alternative for many studies (McClelland, 1974; Walski & Parker, 1974; Almeida et al., 2012). This method has since been adopted by the National Sanitation Foundation of the USA, commonly known as the NSF-WQI. In the geometric mean function illustrated in Eq. 2, the final index is zero if any one sub-index is zero. This characteristic helps to eliminate the eclipsing and ambiguity problem (Liou et al., 2004).
  2 $$\mathrm{WQI=\textstyle\prod^{n}_{i\,=\,1}Qi^{Wi}}$$
  where *Q*_*i*_ = sub-index/transformed parameter *i*, *n* = number of transformed parameters and *W*_*i*_ = weight of the transformed parameter.
(iii) Logical
  The logical aggregation method involves combining the sub-indices using logical operators. The most common logical operators are the minimum and maximum operators, notably used in the Smith index (Smith, 1990).
  Minimum operator function
    The minimum operator is an aggregation function which avoids eclipsing and ambiguity in the final index by using the lowest sub-index values to produce the index value. This function was initially created and applied in New Zealand by Smith (1990) to assess the water quality of lotic systems. However, it was used to evaluate surface water quality in India (Shah & Joshi, 2015). The mathematical expression of the function is given below:
    3 $$WQI = \mathrm{Min} (S_{i}, S_{i \,+ \,1}, S_{i \,+\, 2}, \dots ..S_{subn})$$
    where *S*_*i*_ is the sub-index value for the *i*th parameter and *n* is the number of sub-indices.
  Maximum operator function
    Unlike the minimum operator, the maximum operator aggregation function performs the summation of sub-indices in an increasing scale manner. None of the published WQI models have used this function for aggregation. However, it has been reported to be suited to applications where an index must report if any of the recommended limits are violated (Abassi & Abassi, 2012). The general function of the maximum operator is presented in Eq. 4:
    4 $$I = \mathrm{max}\, (I_{1}, I_{2}, \dots I_{n})$$

In the maximum operator function, *I* assumes the largest of the sub-index values, and *I* = 0 if *I*_*i*_ = 0 for all *i.*

## WQI classification schemes

**Fig. 7:**
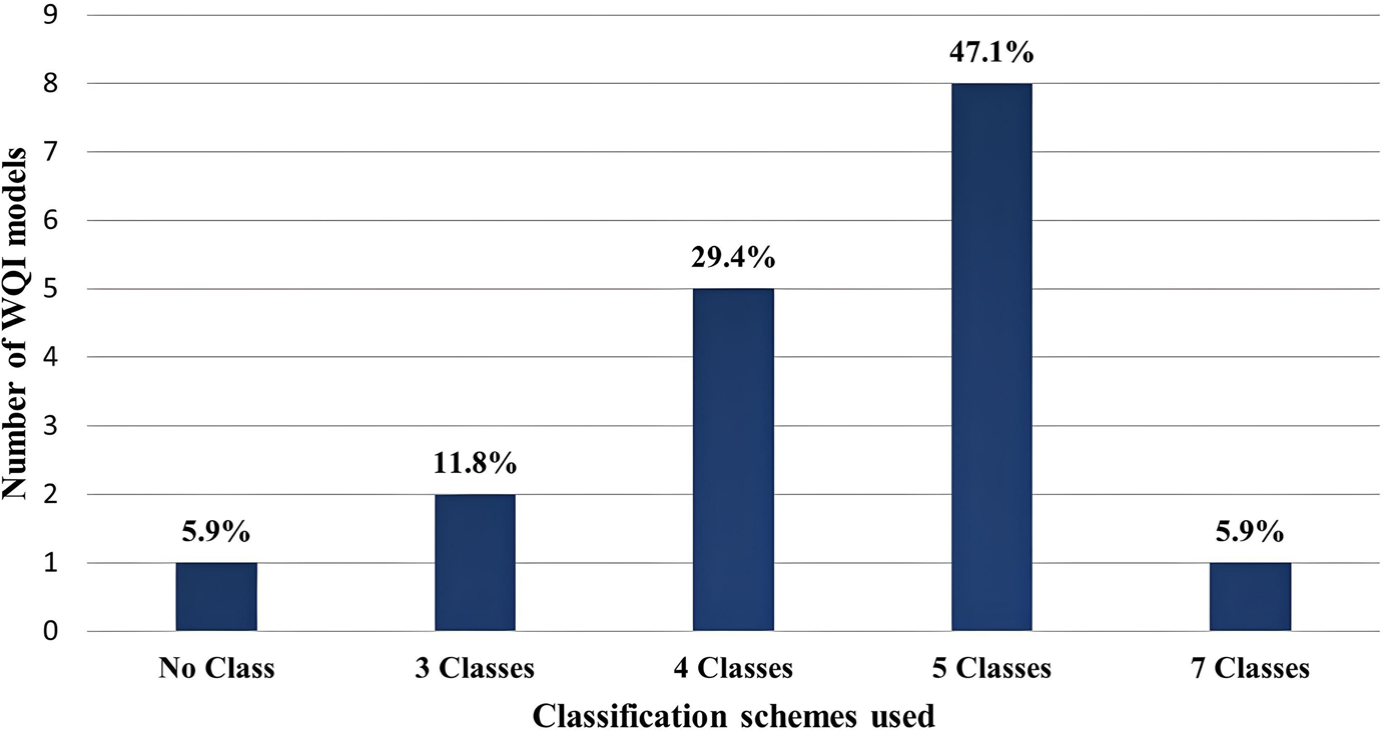
Different classification schemes used among the WQI model to explain the metaphoring problem contributing to WQI uncertainty

The final stage of WQI development involves classifying or categorising water quality based on the index value. These quality classes group the water quality status of water into categories such as “excellent”, “good”, “fair” and “poor”. However, different WQIs use different classification schemes with varying interpretations for the same water quality parameters. Like the other stages in the WQI development, this stage also presents its own distortions that primarily stem out from what has been recently adopted as the “metaphoring problem” (Uddin et al., 2023b). The metaphoring problem denotes that different WQI models employ different classification schemes to explain the WQI value. According to Uddin et al. (2021), the traditional WQI model does not express the actual state of water quality because of the use of various classification schemes, thus prompting model uncertainty and unreliability. Uddin et al. (2022a) advances further ideas that the current WQI model provides ambiguous information to end users of the model and impedes them from responding as quickly as required. Figure 7 gives an illustration of the different classification schemes used among the 17 WQI model, with five classes being the most (47.1%) used, albeit with varying interpretations in selected instances.

It is against this backdrop that recent studies have used more robust, reliable and precise machine learning (ML) techniques such as K-nearest neighbour (KNN), ANN, decision tree, Gaussian Naïve Bayes (GNB), SVN, random forest (RF) and XGB (Najafzadeh & Niazmardi, 2021; Malek et al., 2022; Uddin et al., 2023c). ML classifiers have been effectively used in the recent past to predict the correct classification when assessing water quality (Shakhari & Banerjee, 2019; Najafzadeh & Niazmardi, 2021). In addition, it has recently been proven that the use of XGB yields the most accurate, precise and specific water quality classification schemes (Malek et al., 2022). Furthermore, a recent study piloted in coastal waters evaluated the performance of ML classifiers such as GNB, SVN, KNN and XGB. The authors reported that XGB yielded the most accurate classification for most water quality classes except for the “poor” class (Uddin et al., 2023c).

## Selected common WQI used for evaluation of lotic and lentic ecosystems

### Canadian Council of Ministers of the Environment Index index

This index was initially introduced in Canada in the 1990s as the British Columbia Water Quality Index and used both as a water quality public communication tool and to identify watersheds for priority action. The index was modified and endorsed by the Canadian Council of Ministers of the Environment (CCME) in 2001 and referred to as the CCME WQI (CCME, 2001). Since then, the CCME index has been used in most studies conducted outside Canada in Turkey, India, Iran and Albania (Boyacioglu, 2010; Sharma & Kansal, 2011; Damo & Icka, 2013; Mostafei, 2014) to evaluate the quality of both lentic and lotic ecosystems (Davies, 2006; Giriyappanavar & Patil, 2013; Mostafaei et al*.,* 2014).

(i) *Parameter selection*
  The CCME was designed to be flexible regarding the number of parameters included in the calculation of the final WQI. It, however, requires that minimum of four parameters. This was to accommodate for easy modification to suit local conditions. The CCME does not have a specific parameter selection method, meaning the user can decide on the process according to their needs.
(ii) *Transformation to a standard scale*
  The CCME WQI does not standardise the parameters to a common scale. Instead, it uses standard guidelines or water resource quality objectives.
(iii) *Establishing parameter weights*
  The weighting process is not conducted because there are no sub-indices.
(iv) *Aggregation*
  Aggregation is conducted through a sum root of squares mathematical equation (Eq. 10).

The aggregation function for the CCME is based on scope, frequency and amplitude, denoted by *F*_1_, *F*_2_ and *F*_3_, respectively. It requires that all the parameters be standardised, and the three factors calculated. Scope (*F*_1_) refers to the percentage number of parameters that exceed freshwater ecosystem guidelines and is calculated using the equation below.

5 $$F_{1} =\left(\frac{\text{Number of failed parameters}}{\text{Total number of parametes}}\right)\times 100$$

Frequency (*F*_2_) is the percentage number of tests for each parameter that does not comply with the guidelines. This is calculated using Eq. 5.

6 $$F_{2} =\left(\frac{\text{Number of failed tests}}{\text{Total number of tests}}\right)\times 100$$

Amplitude (*F*_3_) is the extent to which the failed tests exceed the limit guidelines. Unlike *F*_1_ and *F*_2_, the calculation of *F*_3_ involves two steps that determine the excursion and the normalised sum of the excursion (nse). The excursion refers to the number of times a parameter’s test value is greater than that parameter’s objective. This is calculated using Eq. 6. The nse is calculated by adding all the excursions from individual tests from their objectives and dividing them by the total number of tests using Eq. 7. The amplitude (*F*_3_) is then calculated using Eq. 8.

7 $${\mathrm{Excursion}_i}= \left[\frac{\text{Failed test value}\,i}{\text{Objective}\,i}\right]{-1}$$

8 $$\text{nse}= \left(\frac{\textstyle\sum^{n}_{i\,=\,1}\text{excursions}}{\text{number of tests}}\right)$$

9 $$F_{3}= \left[\frac{\text{nse}}{\text{0.01nse}\,+\,0.001}\right]$$

The final CCME index is then calculated using the sum root of the squares of all the factors with Eq. 9.

10 $$\text{CCME WQI}= 100 - \left(\frac{\sqrt{({F_{1}}^{2})\,+\,({F_{2}}^{2})\,+\,({F_{3}}^{2}}}{1.732}\right)$$

In this index, the constant value of 1.732 is used to normalize the resultant values to a 0–100 range where zero (0) depicts poor quality and 100, the best quality of water (Lumb et al., 2011; Rangeti et al., 2015; Sutadian et al., 2016).

The final index is interpreted with value ranges between 0 and 100, with five water quality classes: poor quality 0–44; marginal quality 45–64; fair quality 65–79; good quality 80–94; and excellent quality 95–100.

The scale of application of the CCME WQI has been used in lotic (CCME, 2001) and lentic ecosystems (Giriyappanavar & Patil, 2013).

### Oregon WQI

The Oregon WQI (OWQI) was created to express ambient water quality for recreational uses in 1979 (Dunnette, 1979). The index was discontinued in 1983 because it needed to be more cost-effective (Sutadian et al., 2016). However, Cude (2001) updated the index to interpret the overall quality of water and to communicate the water quality status of Oregon rivers.

(i) *Parameter selection*
  The selection of parameters involved a four-stage elimination process. Stage one involved a thorough literature review of previous WQIs, which accumulated 90 parameters. Stage two involved using rejection criteria: data availability, the significance of parameters and not being present in harmful concentrations. Stage two reduced the parameters from 90 to 30. Stage three involved conducting the Delphi method but only through the Oregon Department of Environmental Quality (ODEQ) members as respondents. This process trimmed down the parameters to 14. The last stage involved conducting another rejection test called redundancy test and impairment categories which reduced the parameters to six, after which two (total phosphorus and temperature) were added in 2001 to a total of eight (Sutadian et al., 2016) based on a better understanding of the importance of these parameters to the streams of Oregon (Cude, 2001).
(ii) *Transformation to a standard scale*
  The current version of the OWQI uses non-linear regression rating curves. In this step, the parameter measurements for each sub-index are converted to a relative quality rating between 10 (worst case) and 100 (ideal).
(iii) *Establishing weights*
  Parameter weights were initially assigned using the Delphi technique on the six parameters. Unequal weights were set as follows: DO (0.4), FC (0.2), pH (0.1), ammonia + nitrate nitrogen (0.1), TS (0.1) and BOD (0.1). However, in the Cude (2001) update, it was argued that unequal weights are only suitable for WQIs, which are developed for a specific use, where some parameters play more important roles than others. Equal weight assignment was then adopted for this index.
(iv) *Aggregation*
  The original aggregation of OWQI was through the weighted arithmetic mean (Eq. 1). However, this aggregation method experienced some eclipsing problems, so the updated version adopted the unweighted harmonic square mathematical formula presented as Eq. 11 below:
  11 $$\text{WQI}=\sqrt{\frac n{\{\sum_{i\,=\,1}^n(Qi)2\}}}$$

The final interpretation of the OWQI is categorised into five classes, namely, excellent (90–100), good (85–89), fair (80–84), poor (60–79) and extremely poor (10–59).

The scale of application for the OWQI is limited to the Oregon streams, and any attempt to apply this WQI to different waterbody types should be done with caution (Cude, 2001). Therefore, the OWQI was only developed to work in local lotic systems.

### National Sanitation Foundation WQI

The NSFWQI was developed in the 1970s in the USA in a process that involved over 100 water quality experts throughout the USA (Brown et al., 1970). Due to its credibility, its direct application or modification has been recorded in most places outside the USA (Mojahedi & Attari, 2009; Benvenuti et al., 2015; Fathi et al., 2018).

(i) *Parameter selection*
  The selection of parameters was based on the consensus of the water quality experts using the Delphi technique. A total of nine parameters were selected from a set of 30 frequently measured parameters in the USA. It was later updated by adding pesticides and toxic elements.
(ii) *Transformation to a standard scale*
  Sub-index generation for the NSFWQI was done through the Delphi technique. The information acquired from the Delphi technique was later used to provide rating curves that represented the guidelines for the parameter in question (Lumb et al., 2012).
(iii) *Establishing weights*
  The Delphi technique applied to decide on the weighting of the selected parameters. The final weights were as follows: DO (0.17), FC (0.16), BOD5 (0.11), temperature (0.10), NO_3_ (0.10), turbidity (0.08), TS (0.07), pH (0.11) and FC (0.16).
(iv) *Aggregation*
  The aggregation method proposed by Brown et al. (1970) took the structure of an additive model, which was later found to be insensitive because one wrong parameter automatically renders the WQI zero. To address this, a multiplicative variation of the NSFWQI was proposed (Brown et al., 1973).

This aggregation method was interpreted as follows: excellent (90–100), good (70–89), medium (50–69), bad (25–49) and very bad (0–24) (Brown & McClelland, 1974).

The scale of application for the NSFWQI was not specified, but the index has been used in lotic systems.

### The Scottish Research Development Department index

The SRDD was initially developed in 1976 for Scotland as a modification or adaptation of the NSFWQI. However, it has been reported in the literature to being in use in other countries to evaluate river basins (Bordalo et al., 2006; Carvalho et al., 2011; Dadolahi-Sohrab et al., 2012).

(i) *Parameter selection*
  Following similar methods as the NSF WQI, the SRDD also used the Delphi technique to select ten parameters with the local water experts as the respondents to the questionnaires (SRDD, 1976).
(ii) *Transformation of parameters*
  The Delphi technique of respondents’ judgment is used to develop the sub-indices of the SSRD index. The possible lowest and highest values of the sub-indices were decided to range between 0 and 100.
(iii) *Establishing weights*
  Parameter weights were assigned using the Delphi technique. The weights for each parameter were as follows: DO (0.18), BOD5 (0.15), free and saline ammonia (0.12), pH (0.09), total oxidised nitrogen (0.08), phosphate (0.08), SS (0.07), temperature (0.05), conductivity (0.06) and *E. coli* (0.01).
(iv) *Aggregation*
  The final aggregation used in the SRDD was a modified additive method like the one used by Bordalo et al. (2006) and Carvalho et al. (2011). This SRDD index had seven classes of water quality, namely, clean (90–100), good (80–89), good water with some treatment (70–79), tolerable (40–69), polluted (30–39), severely polluted (20–29) and water akin to piggery waste (0–19).

The SRDD was adapted from the NSF WQI, so the scale of application was the same as the NSF WQI. Application of the SRDD in lotic systems from reported literature expands to Thailand (Bordalo et al., 2001), Portugal (Carvalho et al., 2011) and Iran (Dadolahi-Sohrab et al., 2012).

### House index

The House index was developed in the late 1980s by House (1989) as a group of four indices that could be used separately or as a hybrid index when more information on river quality was needed. The first index, called general WQI, was for general water quality assessment to indicate river health. The other three, which are the potable water supply index (PWSI), aquatic toxicity index (ATI) and potable sapidity index (PSI), were developed to assess the suitability of potable water supply and toxicity in aquatic and wildlife populations (House, 1989).

(i) *Parameter selection*
  Parameter selection was through interviews with stakeholders in the water sector, including water authorities and bulk water supply water boards. The parameters were chosen by collating the information from the interviews, especially those that the stakeholders routinely monitored. To assess river health, the general WQI had nine parameters: DO, ammoniacal nitrogen, BOD_5_, suspended solids, NO_3_, pH, temperature, chloride and total coliforms. The PWSI, on the other hand, had 13 parameters: DO, ammoniacal nitrogen, BOD_5_, suspended solids, NO_3_, pH, temperature, chloride, total coliforms, fluoride, colour and dissolved iron. The ATI comprised 12 parameters: dissolved copper, polyaromatic hydrocarbons (PAHs) and total pesticides. A similar number of parameters were considered for the PSI and included total copper, zinc, cadmium, mercury, lead, chromium, arsenic, cyanide, phenols, total hydrocarbons, PAHs and total pesticides (House, 1989).
(ii) *Transformation of parameters*
  Rating curves were the preferred method to transform the parameters to a standard scale. The rating curves were developed using the individual parameter’s water quality objectives or compliance criteria. Moreover, when the parameter had two or more quality standards, the median was computed and converted into specific sub-index values (House, 1989; Sutadian et al., 2016).
(iii) *Establishing weights*
  Assigning parameter weights to reflect the relative importance of individual parameters was obtained through a questionnaire survey sent to the operational management participants of the involved stakeholders. The participants were, however, only asked to rank the nine parameters which formed the general WQI and the 13 parameters that formed the PWSI. There was no weight assignment for the ATI and PSI because all the selected parameters had equal importance and were considered harmful to human and aquatic life (House, 1989; Sutadian et al., 2016).
(iv) *Aggregation*
  To compute the final index, a modification of the aggregation method was first used by the SRDD in developing the SRDD index (House, 1989). The final index interpretation is divided into four class categories, namely, class I (71–100), which represents the water of a high quality that is suitable for potable water supply, game fisheries, direct contact recreation and industrial uses; class II (51–70) which represents the water of reasonable quality and suitable potable water supply with conventional treatment, fisheries, indirect contact recreation and most industrial uses; class III (31–50) which represents water that is generally polluted but useful for potable water supply with advanced treatment, indirect contact sport and breeding fish population; and class IV which generally indicates badly polluted water and requiring a sizeable investment in treatment infrastructure but can be used for sewage transport and navigation and non-contact recreational activities (House, 1989).

This review could not trace formal reports of the hybrid House index application or applied with modifications in other regions or water types. However, the general WQI has been used elsewhere outside the UK in river systems (Carvalho et al*.,* 2011). According to recorded literature, the scale of application of the House index has been limited to lotic systems in different regions.

## Discussion

The original architecture of the WQI model by Horton (1965) set the tone for all subsequent WQI models. The methods to select parameters, generate sub-index values, assign parameter weights, aggregate and determine classification schemes have always carried the data distortions of eclipsing, ambiguity and ultimately uncertainty. This has led to the development of multiple WQI models across the world, each presenting its own sources of uncertainty, thus perpetuating the lack of a universal WQI model. The following section seeks to appraise the current issues associated with the WQI development and the current research efforts towards creating a more accurate, robust and acceptable WQI model.

### Issues involved in WQI development

(i) *Parameter selection*
  Indices naturally contain fewer data than actual raw data, and they only incorporate variables deemed necessary for a catchment because of limitations of time, resources and complexity, among others. This may become a problem if certain important variables are left out of the index by mistake because the index will present a contrary picture to the actual water quality (Rangeti et al., 2015; Uddin et al., 2021). For example, according to Zainudin (2010), in Malaysia, they used a WQI which excluded coliform bacteria which is an essential indicator of the microbiological safety of the water.
(ii) *Lack of universal WQI*
  Another critical limitation of WQI is the reported disagreements of the same WQI. For instance, for their evaluation of the surface water quality of the Ganges river, Sharma et al. (2014) used two different scales for the same index. These disagreements have been noted in some cases: when the same index is used but other limits for classes are used (Ramakrishnaiah et al., 2009; Yadav et al., 2010); the same index is used, but the number and type of variables differ. These disagreements add to the need for a universal water quality index.
(iii) *WQI ambiguity and eclipsing*
  Ambiguity in WQI arises due to the selection of parameters and their weightings, which can vary between WQI models. Different models can have different parameter sets and weightings based on their specific objectives and geographical locations. As a result, WQI values can be difficult to interpret and compare across different models and locations (Gupta & Gupta, 2021; Uddin et al., 2021). The authors also discuss several approaches that have been proposed to address ambiguity and uncertainty in WQI. These include incorporating stakeholder perspectives into WQI models, conducting sensitivity analyses to test the robustness of different models and using machine learning techniques to improve the accuracy and predictive power of WQI models (Uddin et al., 2022b, 2023a).
  Eclipsing occurs when a single parameter or group of parameters dominates the calculation of WQI values, causing other parameters to be ignored or given less weight in the final index value. It can also occur due to the subjective selection of parameters and weightings used in WQI models. For example, if a WQI model places greater emphasis on a single parameter such as dissolved oxygen, it may overshadow the impacts of other parameters such as pH or total dissolved solids. This can lead to a misleading assessment of water quality and mask underlying issues (Sutadian et al., 2016; Gupta & Gupta, 2021; Uddin et al., 2021).
  To address the issue of eclipsing, Gupta and Gupta (2021) suggest the use of multi-criteria decision-making approaches in WQI models. Multi-criteria decision-making approaches allow for the simultaneous consideration of multiple parameters and criteria, avoiding the problem of eclipsing. Additionally, other recent studies have suggested the use of sensitivity and uncertainty analyses (Sutadian et al., 2018; Uddin et al., 2022a) to identify the parameters and weightings that have the greatest impact on WQI values. Positive results of the use of these suggestions were revealed by Uddin et al. (2023a) with the IEWQI which presents a novel approach for rating water quality using a combination of fuzzy logic and analytical hierarchy process (AHP) techniques coupled with machine learning to appraise model performance.
(iv) *Uncertainty issues*
  Uncertainty in a model is a fundamental feature related to the model’s specific parameters. As a result, during an analysis of an index uncertainty, much focus is given to how the parameter variation could affect the sub-index values and the final index value (Uddin et al., 2021). Several studies have reported that uncertainty in an index is associated with the various stages of development of the WQI model (Juwana et al., 2016; Seifi et al., 2020). Thus, the purpose of analyzing uncertainty is to determine the source of uncertainty in the whole model and the impact thereof on the final index value (Akhtar et al., 2021). Considering the preceding, the design and development of any WQI model must include a comprehensive uncertainty analysis to improve confidence when applying the model (Sutadian et al., 2018; Uddin et al., 2021). Uddin et al. (2023a) highlighted the importance of sensitivity analysis in quantifying uncertainty and assessing the robustness of any proposed model. Sensitivity analysis can help to identify the parameters and weightings that have the greatest impact on the final water quality rating, allowing for the identification of potential sources of uncertainty and the development of more robust and reliable models. In addition, Uddin et al. (2023c) demonstrated the effectiveness of learning machine learning techniques in improving the predictive accuracy of the WQI model proving that learning machine learning models outperform traditional statistical approaches in terms of predictive accuracy and robustness.
(v) *The metaphoring classification schemes problem*
  This metaphoring problem of water quality classification schemes as referred to by Uddin et al. (2023c) was initially pointed out by Uddin et al. (2021) as the authors discussed the importance of water quality classification schemes which provide a framework for interpreting water quality data and communicating the results to stakeholders and the public. The authors note that different countries and regions have developed their own water quality classification schemes, which can vary in terms of the number and type of parameters considered, the thresholds used to classify water quality and the associated management actions. Considering this, recent studies have highlighted the importance of selecting an appropriate water quality classification scheme that is relevant to the specific context and management goals (Malek et al., 2022; Uddin et al., 2023c). The recent study has demonstrated the persistence of this problem with Fig. 7 showing five different classification schemes for the 17 WQI models analysed. Recent studies have demonstrated that the current WQI model fuels uncertainty (Uddin et al., 2023c) due to high bias and overfitting (Malek et al., 2022). To resolve this, Malek et al. (2022) demonstrated the potential of machine learning algorithms such as multilayer perceptron neural networks, KNN, XGB, SVN, GNB, decision tree and RF to accurately predict water quality classification. The authors revealed that XGB had the highest accuracy and precision. A recent study also supported this finding when they reported that XGB outperformed all other classifier algorithms with 99.9% accuracy (Uddin et al., 2023c).

## Conclusions

Water quality indices are data management tools for communicating complex water quality data to water authorities and the public. They are also used to assess the water quality status or health condition of watersheds at certain times and locations. Although there is yet to be a universally accepted WQI, there is consensus on the development methods. The objective of this review was to analyse water quality indices developed for surface water general evaluation and establish whether the WQIs can be used to evaluate both lentic and lotic ecosystems simultaneously. The following are the main conclusions of the review:

1. Most analysed WQIs are developed in a generic manner involving the five key steps, which include (1) selection of parameters, (2) transformation of parameters to a single scale, (3) assigning parameter weightings, and (4) aggregation of parameters to determine the water quality index value, and (5) determination of the water quality classification schemes. Over 70% of the analysed WQI models used all four steps, and the other 30% used at least two steps. Although most models were developed using the generic method, most are site or region specific and primarily address regional water quality challenges.
2. The selection of parameters is made through subjective methods from experts and local water quality stakeholders. For most WQIs, parameter selection is based on water quality risks, and the high variability confirms this in the number of parameters included in the analysed WQIs. In addition, physical parameters played a significant role as they were used the most, while biological parameters such as faecal coliforms and *E. coli* were used the least. This is an improvement opportunity for WQI models to align the model development process with international standards to improve the acceptability and wide use of a model.
3. Majority of the analysed WQIs used mathematical functions to transform the parameters, while expert opinions were considered the least. Subjectivity has been a constant impediment to the proper adoption of WQI models. Using less subjective methods demonstrates a significant leap towards more robust and impartial methods in the WQI development process.
4. The issues of eclipsing and uncertainty are significant in that they affect the accuracy of a model. These limitations are observed in most WQIs. As such, further studies that seek to reuse an existing WQI model or develop a new one can create a way to score these limitations. This would help users to know how dependable and acceptable the final index is.
5. It has been proven that despite the use of statistical methods such as cluster analysis (CA), factor analysis (FA) and analytic hierarchy process (AHP), WQI models continue to suffer from either eclipsing, ambiguity or uncertainty limitations because natural ecosystems tend to be too complex for these statistical methods.
6. Apart from the CCME, IEWQI and the Hahn indices, the 14 other WQIs are designed only to evaluate one type of freshwater ecosystem. This is a limitation for any institution wishing to solve similar water quality challenges in a different ecosystem type. As such, these indices can be revisited and applied in another system to evaluate if the same effect can be achieved. This will reduce the burden of using more than one index for the same region instead of adapting one index for both ecosystem types.

## Future directions

Models available in the literature have relied on expert opinion for parameter selection and weightage of parameters. This has been a significant contributor to issues of model uncertainty, reliability and adoption by water quality management practitioners and institutions. In addition, the preceding has always been a major limiting factor in the effort towards a universally acceptable WQI. In the more recent studies, such as the development of the West-Java WQI (Sutadian et al., 2018), we observed the effective application of statistical methods in parameter selection (cluster analysis) and assigning of weights (analytic hierarchy process) as a substitute for the more subjective methods of expert opinions while the IEWQI (Uddin et al., 2023a) used fuzzy logic and AHP coupled with machine learning. This improved the acceptability of the West-Java WQI and the IEWQI as reliable tools for managing water resources. Although this is positive, other studies (Elsayed et al., 2021) have argued that natural ecosystems are too complex for these statistical models and suggested models based on machine learning, such as artificial neural networks (ANNs). This is because ANNs can generalise non-linear patterns within a database and solve complex problems (Adnan et al., 2019; Isiyaka et al., 2019). Furthermore, ANNs have been effectively applied to evaluate the accuracy and reduce uncertainty in the final aggregation process (Šiljić Tomić et al., 2018; Uddin et al., 2021, 2023a) and classification schemes (Gupta & Gupta, 2021; Uddin et al., 2023c). Using statistical methods coupled with machine learning techniques requires further exploration in the development process of WQI to eliminate WQI inaccuracies and uncertainties and improve the scope of application.

## Data Availability

The datasets generated during and/or analysed during the current study are available from the corresponding author upon reasonable request.

## Abbreviations

AHP: Analytical hierarchy process
ANN: Artificial neural network
ATI: Aquatic toxicity index
BOD: Biological oxygen demand
CA: Cluster analysis
CCME: Canadian Council for Ministers of the Environment
COD: Chemical oxygen demand
DO: Dissolved oxygen
EC: Electrical conductivity
GNB: Gaussian Naïve Bayes
IEWQI: Irish water quality index
KNN: K-nearest neighbour
NSF: National Sanitation Foundation
NTU: Nephelometric turbidity units
PAH: Polyaromatic hydrocarbons
PCA: Principal component analysis
PRISMA: Preferred Reporting Items for Systematic Reviews and Meta-Analyses
PSI: Potable sapidity index
PWSI: Potable water supply index
RF: Random forest
SRDD: Scottish Research Development Department
SS: Suspended solids
SVN: Support vector machine
TDS: Total dissolved solids
XGB: Extreme gradient boosting
WQI: Water quality index
WQIs: Water quality indices
